# A Setup for Measuring the Centering Error of a Dual-Element Pyroelectric Infrared Sensor Module

**DOI:** 10.3390/s21196684

**Published:** 2021-10-08

**Authors:** Vu Toan Thang, Vu Van Quang, Ngoc-Tam Bui

**Affiliations:** 1 School of Mechanical Engineering, Hanoi University of Science and Technology, Hanoi 100000, Vietnam; thang.vutoan@hust.edu.vn; 2 Shibaura Institute of Technology, College of Systems Engineering and Science, Tokyo 135-8548, Japan

**Keywords:** dual-element PIR sensor, Fresnel lens, centering error, optical axis, modulation, infrared radiation

## Abstract

This paper presents an experimental setup to measure the horizontal centering error of a pre-built pyroelectric infrared (PIR) sensor module, in which a dual-element PIR sensor is aligned at the focal point of a single-zone Fresnel Lens. In the setup, the sensor module was placed facing a modulated infrared radiating source and turned over a range of horizontal angles. The position of the optical axis of the sensor module was determined based on the analysis of the output response of the sensor at turned angles. Thus, the horizontal centering error of the module is defined as the difference between the mechanical axis of the housing and the found optical axis. For the prebuilt sensor module, with the specific available equipment, the measurement of the centering error of the module achieved a resolution of 0.02 degrees.

## 1. Introduction

The Pyroelectric Effect, in which a temperature change in a specific material leads to an output of an electric charge, has been known about for a long time [1]. PIR sensors, which work based on the pyroelectric effect, are featured with their ability to detect intrusions from specific heat sources in their fields of view (FOV) [2]. In practical applications, the PIR sensor is usually assembled with a Fresnel lens—a flat, converted type of plano-convex lens [3]—which plays a role of the objective in order to concentrate infrared (IR) radiation emitted from a heat source onto the sensor surface and, as a result, to magnify the output signal of the sensor. Furthermore, it is worth noting that the dual-element PIR sensor type is used for the purpose of reducing the effect of background temperature change on its output signal [4]. The sensor and the lens usually work in the mid-infrared wavelength range. Therefore, such assembly of the sensor and the lens should be considered as a non-imaging Electro-Optical (EO) system.

**Figure 1:**
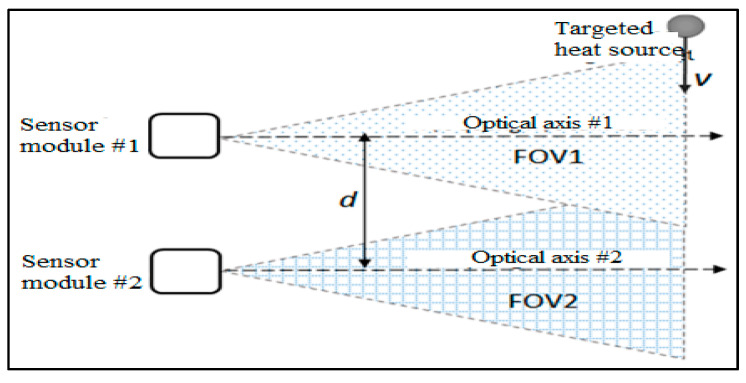
Description of the layout of the vehicle speed measurement system using two PIR sensor modules [10].

Many works have used such PIR sensor modules for various purposes; for example, in commercial applications such as detecting human motion, indoor human tracking and localization [5,6,7], vehicle traffic [8], or even fire alarms [9], etc. In particular, in relation to our recent work [10], a roadside system consisting of two PIR sensor modules, which are supposed to be parallel to each other, was constructed for measuring the speeds of passing vehicles. The layout of our measurement system is illustrated in Figure 1, whereby the moving vehicle’s speed is defined by the length of the time delay between two output signals of those sensor modules when the vehicle passes across their FOV. In such a measurement system, the parallelism of the optical axes of the two sensor modules is critical for the measurement result, as the vehicles may travel at various distances from the measurement system. With the concern of determining the position of the heat source relative to the FOV of such an EO system, in this case, the PIR sensor module, its optical axis, and its centering error are an essential matter.

However, it should be noted that there are very few works using PIR sensor modules that are concerned with the actual FOV and optical axis of the sensor modules. For example, Kastek et al. [11] made a test bed for the evaluation of the angular characteristics of their PIR sensor module by measuring the response of the sensor to a well-controlled moving heat source—a wire heater—across the sensor’s FOV. In another work for indoor human tracking using PIR sensors [4], the authors briefly describe their method to determine the FOV of the sensor, where an incandescent bulb played the role of the heat source, and a chopper was used to modulate infrared radiation to the sensor. Nonetheless, the problem of the centering error of the PIR sensor module has still not been thoroughly resolved, and its analysis should be conducted carefully.

**Figure 2:**
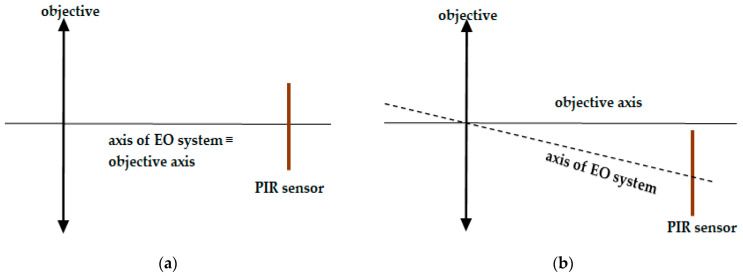
Illustration of the optical axis of a PIR sensor module in perfect alignment (**a**) and in imperfect alignment (**b**).

Moreover, the definitions of the optical axis and, consequently, centering error of a PIR sensor module seem to be difficult to find in the literature. According to [12], the centering error is the angle between the mechanical axis and the optical axis; however, the definition of the optical axis in the same document cannot be applied for EO systems such as the PIR sensor module. Fortunately, Krzysztof Chrzanowski provided a proper definition of the optical axis for non-imaging EO systems [13]: the “optical axis of a non-imaging EO system (IR seeker, receiver of laser seeker, receiver of laser range finder, receiver of laser communication system) is a line that connects the center of the discrete optical detector, the center of a thin lens equivalent to system optics, and a point in the target plane that is the center of the FOV of such a system”. Therefore, in this research, in order to measure the horizontal centering error of the PIR sensor module, we used the definition of an optical axis of the PIR sensor module, demonstrated in Figure 2, which also follows the definition proposed by Krzysztof Chrzanowski for general non-imaging EO systems.

The challenge of determining the position of the optical axis for a prebuilt PIR sensor module is that the module is a non-imaging EO system; thus, the only data are the output voltage signals over time. In addition, there are also many noise sources, such as unexpected radiation noise, Johnson noise, amplifier noise, etc. [1], that might affect the output signal of the sensor. Therefore, a setup for measuring the centering error of a PIR sensor module should be built based on the working principle of PIR sensor and the definition of the optical axis of the module. Moreover, the setup should obtain appropriate measurement sensitivity to define the position of the optical axis of the module with a desired resolution, regarding the presence of noises.

The aim of this study is to construct an experimental setup for defining the position of the optical axis and the centering error. In this setup, an infrared radiation source modulated at a specific frequency is presented and the analysis for parameter settings to enhance the measurement sensitivity is described. The centering error of the PIR sensor module was determined through the sensor output response to the modulated heat source.

Thus, the consecutive sections are constructed as follows. Section 2 describes the working principle of the PIR sensor module which are similar/popular. Section 3 presents the measurement setup with the analysis that points out specific setting parameters that would assist the measurement sensitivity. Section 4 supports the reliability of the proposed setup by providing practical measurements for two PIR sensor modules with specific signal processing methods.

## 2. Dual-Element PIR Sensor Module

**Figure 3:**
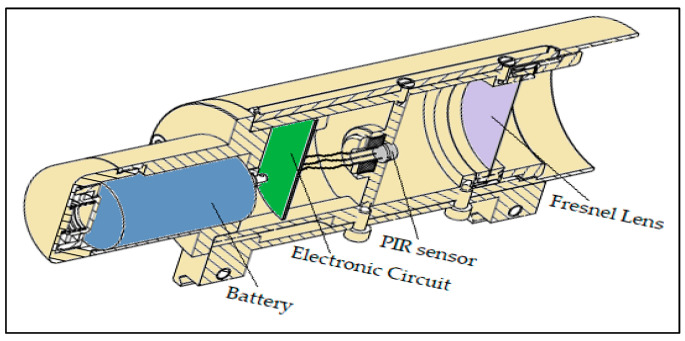
PIR sensor module housing.

The housing of the dual element PIR sensor module was made based on a mechanical design (Figure 3) in order to assemble a Fresnel lens, a dual-element PIR sensor, and electronic board for processing the sensor’s output signal. The lens used in the module was a single-zone Fresnel lens F50.9 from KUBE Electronics AG [14], which played the role of an objective for the EO system. The dual-element PIR sensor was an IRA-S230ST01 sensor from Murata Manufacturing Co., Ltd [15]. Both the sensor and the lens worked with the infrared wavelength range of 5 µm ÷ 15 µm, which features the surface temperature range of −80 °C ÷ 300 °C, due to the Wein displacement law [16]. The PIR sensor was supposed to be placed at the focal point of the Fresnel lens (*f* = 50.9 mm). Such an alignment has appeared in most applications of PIR sensors.

According to this description of the module, a centering error may occur due to various reasons, such as:

Imperfection of the curve of the lens, due to the lens being manufactured as plastic;

Asymmetry of the sensor;

Imperfect placement of the lens and sensor onto the housing.

**Figure 4:**
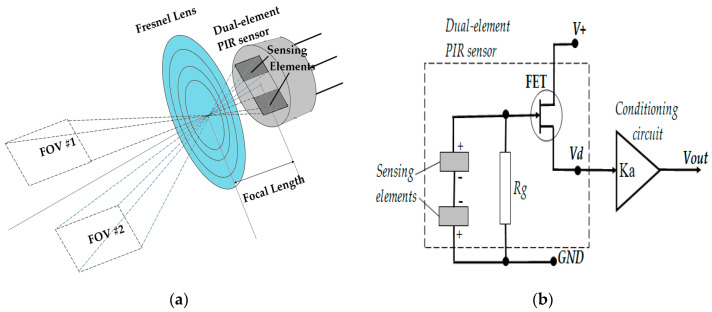
Illustration of fields of view of PIR sensing elements (**a**), and equivalent circuit of PIR sensor module (**b**).

Before describing the setup that was necessary to build, the way in which the specific PIR sensor works should be considered. A dual-element PIR sensor contains two sensing elements that have the same active rectangular areas placed on the same plane, which are systematic regarding the axis of the sensor surface (Figure 4). The formulas that explain how a PIR sensor converts incident radiation into an electrical signal can be found in various literature [2,17]. Overall, the formula of its transfer function in the Laplace domain is found as:

(1) $$\frac{V_{d}\left(s\right)}{\Delta \Phi \left(s\right)}\approx \frac{K_{d}s}{\left(1+\tau_{Th}s\right)\left(1+\tau_{e}s\right)}$$

where ΔΦ—difference of incident radiation powers to two active sensing elements; *V_d_*—output voltage of the sensor; *τ_Th_* and *τ*_e_—thermal and electric time constants that are the main characteristics of the sensor; *K_d_*—the sensor’s amplifier gain. The time constants’ values are usually in the range of a few hundredths of a second to a few seconds, while the amplifier gain is about a few thousand [4].

For the prebuilt PIR sensor module, an external conditioning circuit was built to amplify the sensor’s output voltage and filter the voltage signal within a specific frequency bandwidth. Assuming that the frequency bandwidth is large enough to contain the working frequency bandwidth of the sensor (depending on its time constants), the transfer function of the whole circuit can be written as:

(2) $$\frac{V_{out}\left(s\right)}{\Delta \Phi \left(s\right)}\approx \frac{K_{a}K_{d}s}{\left(1+\tau_{Th}s\right)\left(1+\tau_{e}s\right)}$$

where *K_a_*—amplifier gain of the conditioning circuit.

As can be seen from Equation (2), the output voltage signal of the module characterizes the changing rate of the difference of the incident radiation power to the two sensing elements. On the other hand, the incident radiation power is defined by the heat source within the FOV of each sensing element. When the sensor is aligned at the focal point of the lens, it creates two separate fields of view, corresponding to two sensing elements. The two FOVs are also symmetrical in regard to the optical axis of the sensor module.

Based on the definition of the optical axis for the EO system mentioned in Section 1, and the working nature of the dual-element PIR sensor that is featured in Equation (2), due to the differential component of the transfer function, the following several consequences were taken into consideration:

- If the IR radiation power emitted from sources within the two FOVs of PIR sensing elements is equal all the time, the output voltage amplitude of the sensor module will be zero in an ideal condition (without any noises), or insignificant in reality (with the presence of noises);
- Even in the case that the IR radiation powers emitted from sources within two FOVs are unequal due to their differential component in the transfer function, if the difference in quality between those powers is settled, then the output voltage amplitude will settle to zero in ideal conditions (without noises), or insignificant in real conditions (with noises).

According to those observations for dual element PIR sensor module, a setup for measuring the position of the optical axis of the module based on a modulated infrared radiation source has been built and it is described in the below section.

## 3. Measurement Setup Based on Modulated Infrared Radiation

### 3.1. The Proposed Idea

**Figure 5:**
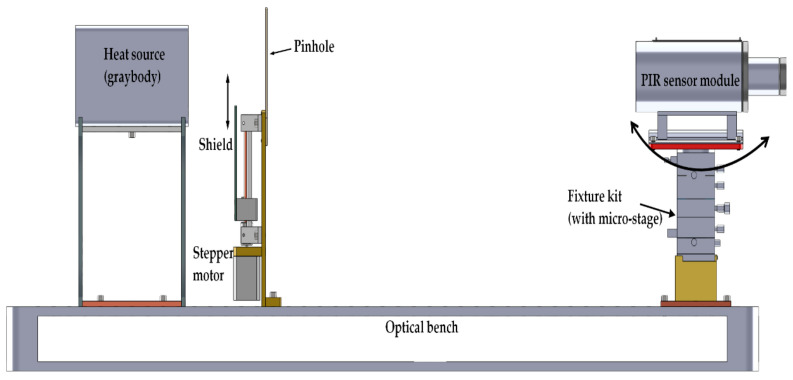
Experimental setup for defining optical axis and centering error of a dual-element PIR sensor module.

The practical setup mentioned in this work was focused on measuring the horizontal position of the optical axis of the EO system (i.e., the PIR sensor module) and contained three particular components (Figure 5):

1. A graybody heat source with a rectangular heating surface, which was intended to be a Lambertian surface;
2. An optical shutter that was designed and made by our research team, with a rectangular pinhole and moving shield which was motorized by a stepper motor controlled by an embedded microsystem;
3. A fixture kit that is based on a manual optical micro-stage, specifically for mounting the sensor module. The micro-stage was able to turn the sensor module around the vertical axis with a resolution of 0.02 degrees.

Due to the limited capability of the equipment, this research only focusses on the horizontal position of the optical axis of the sensor module. However, based on the proposed concept, one could expand the measurement for the positions of the optical axis on other projecting planes.

The three components were aligned on an axis that coincided with the mechanical axis of the sensor module when the module was positioned at a reference angle (Figure 5). The opened area of the pinhole that allowed IR from the graybody to pass through was controlled by the shield, whose movement was controlled by a program on the microsystem. Therefore, the IR radiation power released from the graybody, and incident to the surface of the sensor module was considered to be modulated. Therefore, by adjusting the angular position of the sensor module, once the pinhole was symmetrical around the optical axis of the module, the IR radiation powers in the FOVs of the two sensing elements were equal, regardless of the motion of the shield, and, as a consequence, the amplitude of the output signal stayed at zero in an ideal condition or was insignificant with noises in reality. On the other hand, if the symmetry condition was violated, the output signal would contain a part that has a fundamental frequency similar to the frequency of the field’s motion. The below analysis describes how significant the amplitude of the output signal is regarding the angular position of the optical axis.

### 3.2. Analysis for Measurement Sensitivity

**Figure 6:**
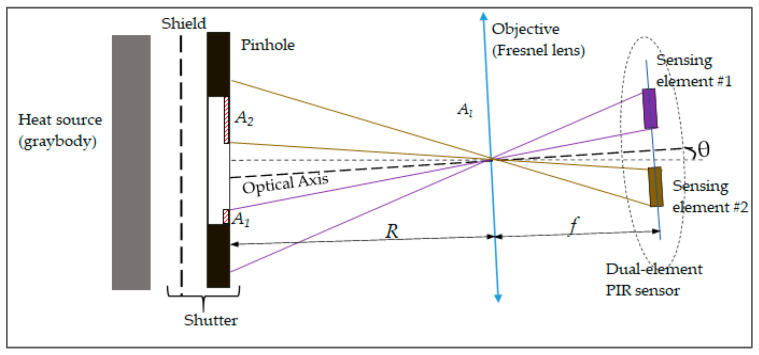
Determination of heat source areas within FOVs of sensing elements in the setup.

The optical axis of the sensor module was positioned at an angle of *θ*, horizontally relative to the axis of symmetry of the pinhole (Figure 6) that was placed at a distance of *R* from the sensor module. Due to the assumption that the radiating source surface is Lambertian, i.e., the radiating energy is equal in all directions, the incident power to each sensing element was calculated as [18]:

(3) $$\Phi_{k}=\frac{\left(L\left(T_{s}\right)A_{s.k}+L\left(T_{b}\right)A_{b.k}\right)A_{l}}{R^{2}}$$

here:

*T_s_*—heat source surface temperature; *A_s_*_.*k*_—the opened area of the pinhole (that lets through infrared radiation from the heat source) within the field of view of the sensing element number *k*;

*T_b_*—ambient background temperature; *A_b_*_.*k*_—the covered area of the pinhole and the background area within the field of view of the sensing number *k*;

*A_l_*—the surface area of the lens, equal to π*D*^2^/4, with *D*—diameter of the lens surface.

*R*—distance from the source to the sensor module;

$L\left(T_{s}\right)$,$\;L\left(T_{b}\right)$—Lambertian functions of the blackbody surface and background temperatures measured in K degrees, defined as definite integrals [16]:

(4) $$L\left(T\right)=\frac{1}{\pi }\int_{\lambda_{1}}^{\lambda_{2}}\varepsilon \left(\lambda \right)\cdot \eta \left(\lambda \right)\cdot \tau \left(\lambda \right)\frac{c_{1}}{\lambda^{5}}\frac{1}{\left(e^{\frac{c_{2}}{\lambda T}}-1\right)}d\lambda$$

here, (*λ*_1_, *λ*_2_) [μm]—working infrared wavelength range of the Fresnel lens and PIR sensing elements; *η*(*λ*)—attenuation due to the atmosphere; *ε*(*λ*)—the emissivity of the heat source object; *τ*(*λ*)—the infrared transmittance of the Fresnel lens; *c*_1_ and *c*_2_ are constants as:

*c*_1_ = 3.741844 × 10^4^ [W∙μm^4^ /cm^2^];

*c*_2_ = 1.438769 × 10^4^ [μm K].

Accordingly, the difference of the incident infrared powers to the two pyroelectric sensing elements was determined as:

(5) $$\Delta \Phi =\left[\left(A_{s1}-A_{s2}\right)\cdot L\left(T_{s}\right)+\left(A_{b1}-A_{b2}\right)\cdot L\left(T_{b}\right)\right]\cdot \frac{A_{l}}{R^{2}}$$

In the case when the angle *θ* was significantly small, the differences of the areas mentioned in Equation (5) were estimated as (Figure 6):

(6) $$A_{s1}-A_{s2}\approx -\left(A_{b1}-A_{b2}\right)\approx -2\cdot h_{R}\cdot R\cdot \tan\theta$$

where *h_R_* is the vertical size of the opened area of the pinhole that does not exceed the value of *h_e_∙R/f* with *h_e_*—vertical dimension of one sensing element; *f*—focal length of the objective. The value of *h_R_* is a function over time whose value depends on the shield’s motion. Supposing that the function is a harmonic one, with the amplitude *H* equal to *h_e_*∙*R/f*, then:

(7) $$h_{R}\left(t\right)=\left[H+H\cdot \sin\left(2\cdot \pi \cdot f_{m}\cdot t\right)\right]/2$$

here, *t*—time variable, *f_m_*—modulation frequency. Thus, the difference in infrared flux ΔΦ is also a function over time, which can be specified as:

(8) $$\begin{array}{cc} \Delta \Phi \left(t\right)= & -\left[L\left(T_{s}\right)-L\left(T_{b}\right)\right]\cdot \frac{A_{l}}{R^{2}}\cdot R\cdot \tan\theta \cdot H\cdot \left[1+\sin\left(2\cdot \pi \cdot f_{m}\cdot t\right)\right]\\ = & -2\left[L\left(T_{s}\right)-L\left(T_{b}\right)\right]\cdot \frac{h_{e}}{f}\cdot A_{l}\cdot \tan\theta \cdot \left[1+\sin\left(2\cdot \pi \cdot f_{m}\cdot t\right)\right] \end{array}$$

With such input radiation power, based on the transfer function represented in Equation (8), the output signal would have the formula:

(9) $$V\left(t\right)=V_{0}\cdot \sin\left(2\cdot \pi \cdot f_{m}\cdot t+\varphi \left(t\right)\right)$$

with *V*_0_—peak voltage amplitude; φ(t)—phase shift. The voltage amplitude is defined as:

(10) $$V_{0}=\frac{K_{a}K_{d}\omega_{m}}{\sqrt{\left(1+\tau_{Th}^{2}\omega_{m}^{2}\right)\left(1+\tau_{e}^{2}\omega_{m}^{2}\right)}}\times \frac{h_{e}}{f}\cdot A_{l}\cdot \left|L\left(T_{s}\right)-L\left(T_{b}\right)\right|\cdot \tan\theta$$

where ω*_m_* = 2π*f_m_* is the angular modulation frequency.

Thus, if the peak voltage amplitude could be measured, the sensitivity of the measurement system would be:

(11) $$\begin{array}{cc} \left|\frac{\partial V_{0}}{\partial \theta }\right|= & \frac{K_{a}K_{d}\omega_{m}}{\sqrt{\left(1+\tau_{Th}^{2}\omega_{m}^{2}\right)\left(1+\tau_{e}^{2}\omega_{m}^{2}\right)}}\times \frac{h_{e}}{f}\cdot A_{l}\cdot \left|L\left(T_{s}\right)-L\left(T_{b}\right)\right|\frac{1}{cos^{2}\theta }\\ \approx & \frac{K_{a}K_{d}\omega_{m}A_{l}\cdot \left|L\left(T_{s}\right)-L\left(T_{b}\right)\right|}{\sqrt{\left(1+\tau_{Th}^{2}\omega_{m}^{2}\right)\left(1+\tau_{e}^{2}\omega_{m}^{2}\right)}}\times \frac{h_{e}}{f} \end{array}$$

The approximation in Equation (11) is held if the value of *θ* is significantly small.

Therefore, the value of the gradient |*∂V*_0_/*∂θ*| that features the measurement sensitivity, can be modified by choosing the following parameters: circuit amplifier *K_d_*, background temperature *T_b_*, heat source surface temperature *T_s_*, and the modulation frequency *f_m_.* It should be noted that even if an increase in the circuit amplifier led to an increase in the gradient |*∂V*_0_/*∂θ*|, it could also increase the noise in the output signal. Moreover, interestingly, the dimensions of the pinhole did not affect the measurement sensitivity, so that they only needed to satisfy the following conditions:

(12) $$\frac{R}{f}w_{g}<W_{p}<\frac{R}{f}\left(2w_{e}+w_{g}\right);\;\mathrm{and}\;\;H_{p}\geq \frac{R}{f}h_{e}$$

where *w_e_*—horizontal dimension of sensing element; *w_g_*—width of the horizontal gap between two sensing elements; *W_p_*—horizontal dimension of the pinhole; *H_p_*—vertical dimension of the pinhole.

From this, we were able to simulate some results by setting the parameters properly. Table 1 gives the values of the parameters needed for the simulation study (it should be mentioned that the given parameters are similar to the real ones):

In particular, in Table 1, the value of the background temperature *T_b_* is preset at 298 K degrees or 25 Celsius degrees. The values of heat source surface temperature *T_s_* and modulation frequency *f_m_* will vary for examining the output signal amplitude.

**Figure 7:**
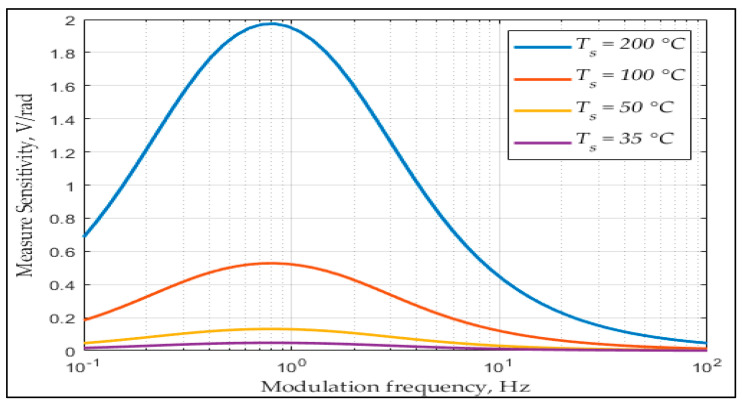
Simulation result for measurement sensitivity over frequency with different settings of heat source temperature.

Figure 7 interprets the dependence of the measurement sensitivity |*∂V*_0_/*∂θ*| on the heat source surface temperature *T_s_* and modulation frequency *f_m_*. It should be pointed out that that the more surface temperature there is, the more IR radiation power is emitted; thus, the measurement sensitivity will increase when the value of the heat source surface temperature *T_s_* increases. Furthermore, the sensitivity will peak when the modulation frequency *f_m_* is of (*τ_Th_ ∙ τ_e_*)^−1/2^/2π.

The analysis and simulation result above provided clues to improve the performance of the setup. The following section shows the experimental result of measuring the centering error of the prebuilt PIR sensor module.

## 4. Experimental Result

**Figure 8:**
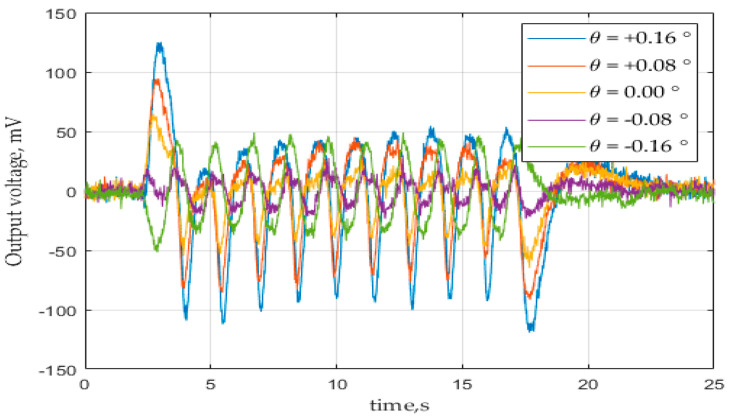
Output voltage signal of PIR sensor module over time at different turned angles.

For conducting experiments to measure the centering error of the prebuilt PIR sensor module, a STM32L476 microcontroller kit was used to sample the sensor’s output voltage signal with a sampling rate *f_S_* of 2000 samples per second, and for transmitting the digitalized data to a computer for further analysis. The heat source temperature was set at *T_s_* = 321 K (48 °C), while the background temperature was set at *T_b_* = 298 K (25 °C). In order to make the modulation as well as the control program for the stepper motor more accessible, the motion of the shield was controlled as a triangular function over time instead of a harmonic one, with the modulation frequency *f_m_* = 0.69 Hz for a period of 1.44 s, and the height amplitude of the opened area at *H =* 40 mm. The heat source temperature was limited due to the available equipment, while the modulation frequency should be set close to the optimal value of (*τ_T_ ∙ τ_e_*)^−1/2^/2π. At each time of measurement, the sensor module was fixed at a horizontal angular position, which was varied every 0.02 degrees by the help of the micro-stage. The output voltage signal of the sensor module was monitored and acquired over a constant duration of time that contained ten periods of the shield motion. Figure 8 illustrates an example of the output voltage signal of a sensor module at different turned angles.

Due to the non-harmonic input and the presence of noises, instead of dealing with the signal amplitude, other quantities (such as signal power, signal magnitude) were taken into consideration. Thus, the determination of the position of the optical axis was found based on one (or both) of two proposed methods:

- Method #1: The angular position of the optical axis is the position where the power spectral density (PSD) of the output signal reaches minimum;
- Method #2: The angular position of the optical axis is the position where the magnitude of the output signal at the modulation frequency reaches minimum.

For method #1, the PSD of the output signal is defined discretely as:

(13) $$P\left(\theta \right)=\frac{1}{N}\overset{N}{\underset{k=1}{\sum }}{\left(V\left(t_{k}\right)\right)}^{2}$$

where *N*—number of samples within the monitored duration; *V(t)*—output voltage at moment *t*; and *t_k_* (*k* = 1…*N*)—discrete moments sampled within the duration.

For method #2, the magnitude of the signal at the frequency at the modulation frequency was estimated using the Discrete Fourier Transform for the series {*V*(*t_k_*)}*_k_*_=1…*N*_:

(14) $$M\left(\theta \right)=DFT\;\left[{\left\{V\left(t_{k}\right)\right\}}_{k=1\ldots N},\;f_{m}\right]$$

At a glance, the two methods would have taken a similar calculation complexity over *N* multiplications. While method #1 appeared simple, method #2 may have given a better result due to the elimination of noises at other frequencies than the modulation frequency.

A number of measurements of the optical axis position at various turned angles have been conducted repeatedly for two (02) PIR sensor modules. Table 2 shows the result of their optical axis position using the two proposed signal processing methods. The values of quantities *P* and *M* presented in Table 2 are calculated as averages of measurement results from 10 measurements at each single turned angle.

**Figure 9:**
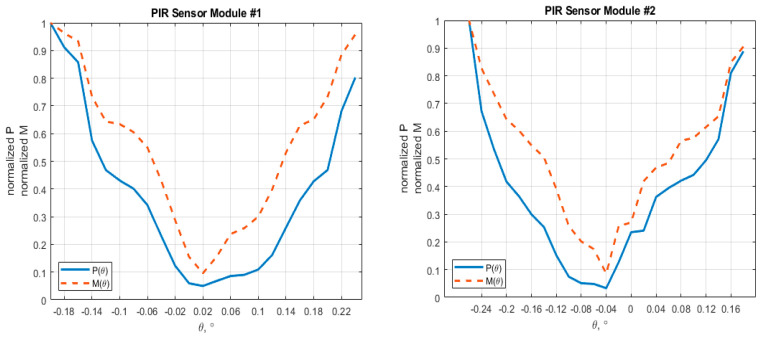
Experiment results of normalized PSD and magnitude at modulation frequency over different turned angles for two PIR sensor modules.

Figure 9 illustrates the normalized curves of those functions over the specific range of turned angular positions.

Based on the data presented in Table 2, the two methods gave the same results when determining the angular positions of the optical axes of the two sensor modules relative to their mechanical axes. Thus, for sensor module #1, its horizontal centering error was determined to be +0.02, and for sensor module #2, −0.04. In order to evaluate the effectiveness of the proposed methods, one should notice the gradients of the curves at local points of the minimum. For PIR sensor module #1, the gradients are 0.49 deg^−2^ and 2.97 deg^−2^ for the normalized P and M curves, respectively. For PIR sensor module #2, they are 0.77 deg^−2^ and 4.43 deg^−1^, respectively.

## 5. Conclusions

A setup for defining the centering error of a PIR sensor module, which is considered to be an Electro-Optical system, has been presented in this paper. The setup was mainly built by constructing a modulated heat source that consisted of a standard radiating surface, and a shutter with a programmable motorized field. An analysis that addressed the setting conditions and parameters, which would support the measurement sensitivity, was also described in this study. Owing to the experimental results, method #2, using DFT, presented a more reliable result due to the local gradient of the peak. With the available equipment, measurements were conducted with a resolution of 0.02 degrees. Based on the proposed setup, the analysis, and the practical measurement results presented in this work, it is suggested that a more accurate measurement for the centering error of similar PIR sensor modules could be made in better conditions. Examples of these are: better thermal isolation for the whole setup, a higher heat source temperature, improved input modulation (in terms of frequency, to make it closer to the optimal frequency, as well as waveform), more accurate and higher-resolution turning equipment, etc.

## Figures and Tables

**Table 1:** Parameter values for simulation.

Parameter	Value	Unit
Sensing element dimension, *h_e_* × *w_e_*	2 × 1	mm × mm
Gap between sensing elements, *w_g_*	1	mm
Thermal time constant, *τ_Th_*	0.5	s
Electrical time constant, *τ_e_*	0.08	s
Sensor amplifier gain, *K_d_*	2200	V∙s/W
Circuit amplifier gain, *K_a_*	20	(none)
Lens diameter, *D*	50	mm
Objective focal length, *f*	50.9	mm
Dimensions of pinhole, *H_P_* × *W_P_*	45 × 45	mm × mm
Distance, *R*	850	mm
Working wavelength range, *λ*_1_ ÷ *λ*_2_	5 ÷ 12	µm
Heat source emissivity, *ε*	0.95	(none)
Atmosphere attenuation, *η*	1	(none)
Transmittance of the lens, *τ* ^1^	0.75	(none)
Background temperature, *T_b_*	298	K

**Table 2:** Experimental results.

Tested Module	Turned Angle, ^°^	P, mV^2^	M, mV
Sensor Module #1	+0.24	977.71	41.66
	+0.22	829.40	38.48
	…	…	…
	+0.06	208.74	10.32
	+0.04	166.02	6.78
	+0.02	60.53	4.16
	0.00	144.98	6.75
	−0.02	189.53	12.49
	…	…	…
	−0.20	1218.17	43.49
	−0.22	1449.07	48.41
Sensor module #2	+0.18	2252.51	60.74
	+0.16	2054.72	56.90
	…	…	…
	0.00	610.54	18.13
	−0.02	322.23	17.26
	−0.04	84.01	5.74
	−0.06	122.90	11.68
	−0.08	189.24	13.52
	…	…	…
	−0.24	1704.31	55.47
	−0.26	2535.29	67.07

## Data Availability

This study did not report any data.
